# A curated dataset for hate speech detection on social media text

**DOI:** 10.1016/j.dib.2022.108832

**Published:** 2022-12-17

**Authors:** Devansh Mody, YiDong Huang, Thiago Eustaquio Alves de Oliveira

**Affiliations:** Department of Computer Science, Faculty of Science and Environmental Studies, Lakehead University, Ontario, Canada

**Keywords:** Hate speech, Cyberhate, Cyberbullying, Natural language processing, Online Hate

## Abstract

Social media platforms have become the most prominent medium for spreading hate speech, primarily through hateful textual content. An extensive dataset containing emoticons, emojis, hashtags, slang, and contractions is required to detect hate speech on social media based on current trends. Therefore, our dataset is curated from various sources like Kaggle, GitHub, and other websites. This dataset contains hate speech sentences in English and is confined into two classes, one representing hateful content and the other representing non-hateful content. It has 451,709 sentences in total. 371,452 of these are hate speech, and 80,250 are non-hate speech. An augmented balanced dataset with 726,120 samples is also generated to create a custom vocabulary of 145,046 words. The total number of contractions considered in the dataset is 6403. The total number of bad words usually used in hateful content is 377. The text in each sentence of the final dataset, which is utilized for training and cross-validation, is limited to 180 words. The generated contractions dataset can be used for any projects in the area of NLP for data preprocessing. The augmented dataset can help to reduce the number of out-of-vocabulary words, and the hate speech dataset can be used as a classifier to detect hate or no hate on social media platforms.

**Specifications table**

**Table:** 

Subject	Artificial Intelligence
Specific subject area	A curated dataset comprising emojis, emoticons, and contractions bundled into two classes, hateful and non-hateful, to detect hate speech in text.
Type of data	Text
How the data were acquired	The hate speech dataset was curated from various sources. The sources were combined into one extensive dataset and labeled into two classes hateful and non-hateful. After combining the data, additional preprocessing steps were incorporated before generating the final dataset for training and cross-validation. Contractions were expanded, and emoticons and emojis were mapped to text.
Data format	Annotated, Analysed, Filtered Data
Description of data collection	The raw data for hate speech detection were collected from 18 sources, and contractions data were collected from 8 sources. To generate a raw dataset, only two classes, hateful and non-hateful, were extracted from the data. This raw dataset was cleaned and pre-processed by dropping duplicates, language correction, removing unwanted characters, converting emojis, emoticons and numbers to text, and expanding contractions.
Data source location	The raw data used for hate speech detection is collected from the below-mentioned sources: • [1]https://github.com/t-davidson/hate-speech-and-offensive-language • [5]https://huggingface.co/datasets/classla/FRENK-hate-hr • [6]https://huggingface.co/datasets/viewer/?dataset=hatespeech18 • https://data.mendeley.com/datasets/jf4pzyvnpj/1 • https://www.chatcoder.com/data.html • https://www.kaggle.com/mrmorj/hate-speech-and-offensive-language-dataset • https://www.kaggle.com/usharengaraju/dynamically-generated-hate-speech-dataset • https://www.kaggle.com/arkhoshghalb/twitter-sentiment-analysis-hatred-speech • https://www.kaggle.com/surekharamireddy/malignant-comment-classification • https://www.kaggle.com/munkialbright/classified-tweets • [7]https://hasocfire.github.io/hasoc/2019/dataset.html • [8]https://github.com/cardiffnlp/tweeteval • https://github.com/tazeek/BullyDetect • https://www.kaggle.com/c/detecting-insults-in-social-commentary/data • [9]https://github.com/HKUST-KnowComp/MLMA_hate_speech/blob/master/hate_speech_mlma.zip • https://www.kaggle.com/vkrahul/twitter-hate-speech • https://www.kaggle.com/pandeyakshive97/hate-speech-dataset • https://www.kaggle.com/eldrich/hate-speech-offensive-tweets-by-davidson-et-al The data to used to create the contractions dictionary is collected from the below-given sources: • https://en.wikipedia.org/wiki/Wikipedia:List\_of\_English\_contractions • https://stackoverflow.com/questions/19790188/expanding-english-language-contractions-in-python • https://github.com/kootenpv/contractions/tree/master/contractions/data • https://7esl.com/contractions-list/ • https://www.eslbuzz.com/popular-contractions/ • https://www.netlingo.com/acronyms.php • https://grammar.yourdictionary.com/slang/texting-slang.html • https://www.noslang.com/dictionary/
Data accessibility	Repository name: Mendeley Data Data identification number (DOI number): 10.17632/9sxpkmm8xn.1 Direct link to the dataset: https://data.mendeley.com/datasets/9sxpkmm8xn/1

## Value of the Data

• This dataset is useful for training machine learning models to identify hate speech on social media in text. It reflects current social media trends and the modern ways of writing hateful text, using emojis, emoticons, or slang. It will help social media managers, administrators, or companies develop automatic systems to filter out hateful content on social media by identifying a text and categorizing it as hateful or non-hateful speech.
• Deep Learning (DL) and Natural Language Processing (NLP) practitioners can be the target beneficiaries as this dataset can be used for detecting hateful speech through DL and NLP techniques. Here the samples are composed of text sentences and labels belonging to two categories “0″ for non-hateful and “1″ for hateful.
• Additionally, this data set can be used as a benchmark data set to detect hate speech
• The final preprocessed data set is neutralized in such a way that it can be used by anyone as it doesn't include any entities or names which can have an impact or cyber harm on the user that generated the content. Researchers can take advantage of the pre-processed dataset for their projects as it maintains and follows the policy guidelines.
• The dataset presented here provides an alternative to smaller, more specialized datasets like the ones in [2,3], and [4].

## 1 Objective

Nowadays, social media text comprises slang, emojis, and emoticons. The conceived dataset aims to handle these diverse input types embedded in the text and enable the training of more effective Artificial Intelligence Models to identify hate speech. The previously available datasets from single sources were insufficient to represent the variations in hate speech content, so the new dataset aggregates and annotates data collected from various sources.

## 2 Data Description

Dataset used to identify hate speech has 451,709 samples in total. 371,452 of these are non-hateful speech, and 80,250 are hateful speech. An augmented data to generate vocabulary has 726,129 samples. The number of words in the vocabulary is 145,046. The number of entries in the contraction dictionary is 6403. The total number of hateful bad words is 377. The length of the text is set to a maximum of 180 words in our final dataset. Table 1 brings a thorough description of the data.

**Table 1:** Columns in the dataset and its descriptions.

Column	Description

Content	This column contains the input text. For example: Then, we added more interesting complex relationships. implied that, after time, one thing would lead to another. means that “I'll be able to talk soon.” We created a scale for asking “How do you feel?”:

Label	This column contains the input label 0 and 1. “0″ means non-hateful “1″ means hateful

The dataset made available also contains a contraction dictionary used to process the raw data into the final pre-processed dataset. The contraction dictionary has the format presented in Table 2.

**Table 2:** Sample contractions and their possible expanded forms from the contractions dictionary.

Contraction	Possible expanded forms

``ain't''	``am not / are not / is not / has not / have not''
``aren't''	``are not / am not''
``can't''	``cannot''
``can't've''	``cannot have''
``cause''	``because''

The dataset also contains a list of bad words usually employed in hateful content, e.g., Fuck, cum and others.

In the raw data, the proportion of hateful and not-hateful samples is 17.8% and 82.2%.

## 3 Experimental Design, Materials and Methods

The final pre-processed dataset containing the aggregated and annotated sentences was acquired by applying the following method:

1. In the data made available in the online repository, the *0_RawData* folder contains data collected from the different sources listed in the specification table to assemble a dataset of sentences.
2. A dictionary of contractions and a list of profanities commonly used in the English language by internet users is made available in the folder *1_ContractionProfanitiesEnglish*.
3. The preprocessed dataset can be found in the *2_PreprocessedData* folder with sentences generated by the following process:
  (a) Emojis and emoticons from the raw data were converted to text and hyperlinks, user mentions, multiple spaces new line characters were removed from the sentences.
  (b) After all these elements were removed, contractions found in the text were then expanded. Grammatical errors were corrected using the word mover's distance between sentences generated from the multiple possibilities for expanded contractions using the Google News Word2Vectors [10] and the open-source Gensim model [11].
  (c) The date and time occurrences were removed.
  (d) Accented numbers and characters were also removed, along with the ampersand symbols in the beginning of some words.
  (e) The remaining numbers were converted to words.
  (f) Special characters (_"\-;%()|+&=*%.,!?:#$@[]/) were then removed from the text and an intermediate file was generated (*preprocessed_data_yidong_devansh.csv*)*.*
  (g) From this file, the misspelled profanities were replaced with the correctly spelled versions based on the *Profanities.csv* file to generate the final preprocessed dataset (*final_preprocessed_data_yidong_devansh.csv*)*.*
  (h) Duplicated entries in the *final_preprocessed_data_yidong_devansh.csv* were also removed before data augmentation and class balancing procedures.
4. In the *3_DataAugmentationAndBERTVocab* folder, the raw datafile *Final_Y_D_data.csv* was used to generate a balanced version of the dataset (*YD_aug_data_balanced.csv*) by *a*) undersampling the class with the majority of samples (non-hateful class); and *b*), augmenting sentences from the hateful class using the contextual word embeddings from BERT models [12] with substitution and insertion methods as well as the synonym augmentation using WordNet embeddings [13].
5. In the *4_PretrainedBERT* folder, a custom BERT tokenizer, vocabulary, and configuration are made available to NLP practitioners.
6. The training data and validation folds for a 5-Fold cross-validation scheme can be found in the *5_TrainValidationFolds* folder. After the preprocessed dataset is created, the samples are randomly shuffled and partitioned into five different folds. These folds are stratified to contain a roughly balanced number of samples for both classes.
7. 6_MissclassifiedBERT2DataFolds Contains sentences that were misclassified by the HS-Bert Model and their true labels. The HS-BERT is a fine-tuned BERT model for hate speech detection. This model was trained on the 5-Fold Cross Validation data. The samples misclassified during training and validation were selected to compose a new data folder containing sentences that may be hard for such models to classify. Other researchers may use these sentences to improve their models in active learning schemes.

## Ethics Statements

The data in the manuscript have been acquired via web scraping. The Terms of service (ToS) for all web resources listed in the specification table and used in the curated dataset allow the scrapping and distribution of data. In terms of copyright, the data curated belongs to web resources and not the users of those resources. No news website was used in the curated dataset. This dataset protects the privacy of individuals. No one's personal information was taken during the data collection. Identities of entities were removed if they occurred in the dataset, and the dataset was neutralized to comply with legal and ethical standards and policy regarding the use of social media data for research purposes. The purpose of the task is to detect hate speech, not to target a particular user or group of users. The dataset collected was curated from publicly available sources. The present work does not contain data scrapped directly from social media platforms (e.g., Twitter and Facebook); thus, it does not violate the scrapping policies of such platforms.

## CRediT Author Statement

**Devansh Mody:** Conceptualization, Methodology, Software, Validation, Investigation, Resources, Data Curation, Writing – original draft, Writing – review & editing; **YiDong Huang:** Conceptualization, Methodology, Software, Validation, Investigation, Resources, Data Curation, Writing – original draft, Writing – review & editing; **Thiago Eustaquio Alves de Oliveira:** Methodology, Validation, Investigation, Resources, Writing – original draft, Writing – review & editing, Supervision, Project administration, Funding acquisition.

## Declaration of Competing Interest

The authors declare that they have no known competing financial interests or personal relationships that could have appeared to influence the work reported in this paper.

## Data Availability

A Curated Hate Speech Dataset (Original data) (Mendeley Data).
